# Adapting a trapped ion mobility spectrometry-Q-TOF for high *m/z* native mass spectrometry and surface-induced dissociation

**DOI:** 10.1021/acs.analchem.4c03557

**Published:** 2025-02-16

**Authors:** Yu-Fu Lin, Benjamin J. Jones, Mark E. Ridgeway, Erin M. Panczyk, Arpad Somogyi, Desmond A. Kaplan, Ila Marathe, Sangho Yun, Karen A. Kirby, Stefan G. Sarafianos, Arthur D. Laganowsky, Melvin A. Park, Vicki H. Wysocki

**Affiliations:** 1. Department of Chemistry and Biochemistry, The Ohio State University, Columbus, OH 43210, United States; 2. Native MS Guided Structural Biology Center, The Ohio State University, Columbus, OH 43210, United States; 3. Bruker Daltonics Inc., Billerica, MA 01821, United States; 4. KapScience LLC, Tewksbury, MA 01876, United States; 5. Center for ViroScience and Cure, Laboratory of Biochemical Pharmacology, Department of Pediatrics, Emory University School of Medicine and Children’s Healthcare of Atlanta, Atlanta, GA 30307 United States; 6. Department of Chemistry, Texas A&M University, College Station, TX 77840, United States

**Keywords:** Native Mass Spectrometry, Trapped Ion Mobility Spectrometry, Surface-induced Dissociation

## Abstract

Native mass spectrometry (nMS) is an increasingly popular technique for studying intact protein quaternary structure. When coupled with ion mobility, which separates ions based on their size, charge, and shape, it provides additional structural information on the protein complex of interest. In this study, we present a novel prototype TIMS (trapped ion mobility spectrometry)-Quadrupole-SID (surface-induced dissociation)-Time of Flight, TIMS-Q-SID-TOF, instrument for nMS. The modifications include changing the TIMS cartridge from concave to convex electrode geometry and operating TIMS at 425 kHz RF to improve the trapping efficiency for high mass-to-charge (*m/z*) ion mobility analysis, such as 3 and 4 MDa hepatitis B virus capsids. The quadrupole radiofrequency driver was lowered to 385 kHz, which extends the isolation range from 3,000 to 17,000 *m/z* and allows isolation of a single charge state of GroEL at 16,200 *m/z* with an isolation window of 25 *m/z*. Finally, a 6-mm thick, 2-lens SID device replaced the collision cell entrance lens. SID dissociated 801 kDa GroEL into all combinations of subcomplexes, and the peaks were well-resolved allowing for confident assignment of product ions. This is the first time a novel prototype timsTOF Pro for nMS has been introduced with high resolving power ion mobility separation coupled to high *m/z* quadrupole selection and SID for protein complex fragmentation with product ion collection and detection across a broad *m/z* range of 1,500 to 40,000.

## Graphical Abstract

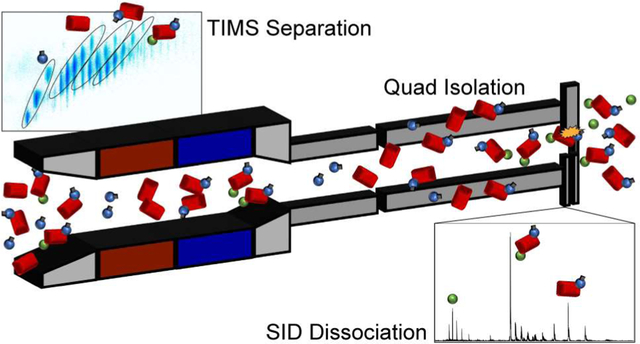

## Introduction

Native mass spectrometry (nMS), particularly in combination with unique activation methods and ion mobility spectrometry (IM), has proven a powerful structural biology technique for investigating topology, stoichiometry, and conformations of proteins and their complexes. Trapped ion mobility spectrometry (TIMS) is a high-resolution ion mobility spectrometry technique that was first introduced by Park and co-workers in 2011.^1,2^ Unlike traditional drift tube ion mobility cells (DTIMS) which use ions flying through a stationary gas in the drift tube, TIMS separates ions by using an opposing electric field and a moving column of gas to measure the analyte’s ion mobility.^3^ TIMS has been coupled to Q-TOF and FT-ICR mass spectrometers and is widely applied in proteomics and metabolomics, including cross-linking mass spectrometry.^4–8^ Recently, Panczyk et al. demonstrated that a commercial TIMS-Q-TOF can perform nMS analysis on protein complexes up to 60 kDa with TIMS.^9^ Borotto and co-workers showed that a commercial TIMS-Q-TOF could also characterize protein structure up to 66 kDa by top-down sequencing and collision-induced unfolding by activation within the TIMS cartridge.^10,11^ Bleiholder and co-workers used a modified tandem TIMS-Q-TOF to characterize the structure of a glycoprotein, avidin, and small proteins.^12,13^ Fernandez-Lima and co-workers reported that a modified convex TIMS cartridge with a low radio frequency (RF) driver improves the ion mobility analysis of TIMS up to 800 kDa with a single peak resolution (R_p_) of 85 compared to DTIMS with an R_p_ of 60 for protein complexes around or above 200 kDa.^14–16^ Because nMS users and applications are continuing to expand^17–21^, and coupling it with IMS provides additional protein and protein complex structural information^22–24^, we aimed to adapt a TIMS^1–3,25^ platform to perform nMS for studying and understanding large, complex protein structures up to the mega-Dalton (MDa) range with a high energy deposition tandem mass spectrometry (MS/MS) activation method, surface-induced dissociation (SID).^26–29^ The Wysocki lab has extended SID development to investigate protein structure and subcomplex connectivity for macromolecules.^27,30–32^ SID cleaves protein complexes at their weakest non-covalent interfaces and generates subunits with a charge state approximately proportional to the relative fragment ion mass.^30,33^ Currently, nMS has been applied to measure the structures of macromolecules in the MDa mass range, such as adeno-associated virus capsids (3.5 – 5 MDa), antibody-bound capsids species (5 – 17 MDa), and oligomerized 20S proteasome (0.7 – 9.6 MDa), which are important for producing biotherapeutics.^34–37^ The Wysocki lab has demonstrated that SID is currently the only activation method capable of extensively dissociating these MDa biomolecules, making it a valuable tool for investigating the structure of AAVs.^38^

Here, we modified a commercial TIMS-Q-TOF mass spectrometer to study the structures of high molecular weight (MW) protein complexes. The modifications include changing the TIMS cartridge geometry to convex, lowering the RF frequency for both the TIMS cartridge and quadrupole, and installing a 2-lens SID device at the collision cell entrance.^39^ With these novel modifications, the application of nMS with ion mobility and SID on this prototype platform ranges from 50 kDa up to at least 4 MDa. This work presents the first demonstration coupling a high-mass TIMS cartridge, high-mass quadrupole, and unique activation technique (SID) to probe biomolecular structure on a mass spectrometry platform commercially designed for non-structural bottom-up proteomics experiments.

## Experimental Section

### Materials and Sample Preparation.

More details on materials and selected protein complexes can be found in the Supporting Information. All protein complexes were buffer exchanged into 200 mM ammonium acetate using size exclusion chromatography spin columns (Bio-Rad) with a 6 kDa mass cutoff. Complexes were charge-reduced using 20% (v/v) triethylammonium acetate (TEAA) because it has been observed that lower charge state protein complexes’ ions generally maintain more native-like structures, and SID dissociates these charge-reduced protein complexes into subcomplexes, which is also retain native-like structures.^40,41^ The sample stock solution concentrations were measured using a Thermo Scientific NanoDrop 2000C and diluted in the range of 0.7 to 3 μM per complex with or without charge reduction. For T=3 and T=4 hepatitis B virus (HBV) capsids, the concentration was 6 μM per monomer and 4.8 μM per monomer after charge reduction. For the RAS-SOS complex analysis, 3 μM SOS was mixed with 2 μM HRasWT-GTP and immediately introduced into the mass spectrometer via a pulled glass capillary.

### Instrumentation and experiments.

Experiments were conducted on a timsTOF Pro (TIMS-Q-TOF) (Bruker Inc., Billerica, MA), modified for the research presented here. A 385 kHz RF frequency quadrupole driver was installed to extend the quadrupole isolation range from 3,000 *m/z* with a commercial 1 MHz RF frequency quadrupole driver to 17,000 *m/z*. Here, we used ESI Tuning Mix and protein complexes (charge-reduced streptavidin, C-reactive protein, pyruvate kinase, and glutamate dehydrogenase) for quadrupole calibration up to 12,000 *m/z*. With the described modifications, the quadrupole can isolate a single charge state at 16,200 *m/z* with an isolation width of 25 *m/z* (Figure S1). Table S1 shows the default instrument parameters of the commercial instrument for analyzing small molecules in column two and the recommended settings for proteins/protein complexes in columns 3–5.

In general operation, the “TIMS In” pressure, which is the TIMS cartridge entrance pressure, was set to 2.2 mbar. The “Accumulation time” and “Ramp Time” of the TIMS cartridge were adjusted to 100.0 ms and 1,000.0 ms (10.00 % Duty Cycle), respectively. The “Funnel 1 RF” was 300.0 volt peak-to-peak (V_pp_). The “Collision Cell In”, which determines the *m/z* transferring range, was 150.0 V. The “Pre Pulse Storage”, a delay for collecting ions between “Transfer Time” and “TOF Pulser On”, was 35.0 μs. The “Transfer Time” is the time corresponding to transmitting ions from the collision cell to the TOF stage and was set to 140.0, 320.0, and 520.0 μs depending on the size of the protein complex. Increasing the “Transfer Time” can transfer larger ions to the TOF. The “Collision Gas Flow Rate” was 35.0 % for the ESI Tuning Mix (Agilent, Santa Clara, CA) and 88.0 % for protein complexes. The “Delta Values (*Δ*t1 to *Δ*t6)”, shown on Figure S2, were used to control the TIMS cartridge. The *Δ*t2, *Δ*t4, and *Δ*t6 were change from −120, 100, and 100 V to −90, 70, and 10 V, respectively, for nMS experiments. More details of the typical settings for nMS can be found in the Supporting Information (Table S1).

All samples were loaded into in-house pulled borosilicate glass capillaries and ionized using a custom Bruker nanoelectrospray ionization source. The ionization is performed by bringing a platinum wire into contact with the sample solution and setting the capillary voltage to (0.8 – 1.0 kV). The instrument was mass calibrated using ESI Tuning Mix. The SID experiments were conducted with or without single charge state isolation using the quadrupole, as noted in the Results section, and the SID potentials were varied appropriately to initiate the fragmentation of different sizes of chosen analytes (Tuning and operation of the SID device is shown in Table S2 and S3). Previously, cesium iodide and Agilent ESI Tuning Mix has been reported to use as calibrant for TIMS to calculate collision cross-sections (CCSs) of protein complexes.^7,12,42^ Here, the protein complexes’ CCSs were calculated using the measured mobilities (K) determined after calibrating the TIMS cartridge with ESI Tuning Mix under experimental conditions. The typical acquisition time for lower *m/z* complexes was 2 to 5 minutes. For HBV and GroEL SID results, the acquisition time was 30 minutes. Although 0.5 minutes of acquisition time was sufficient to identify the SID products, with longer acquisition times, the spectra have a higher signal-to-noise ratio (S/N), which provides higher quality spectra (Figure S3). The instrument was operated using Bruker’s otofControl 6.2 software, and the data were analyzed using DataAnalysis 5.3. The center and full width half maximum height (FWHM) of peaks were determined using MATLAB R2020a. The single peak resolution was defined as the ratio of the peak center location divided by its FWHM for both ion mobility (^IM^R_p_, (1/K_0_)/*Δ*(1/K_0_)) and *m/z* (^*m/z*^R_p_, *m/z*/*Δm/z*).

## Results and Discussions

**Figure 1.**
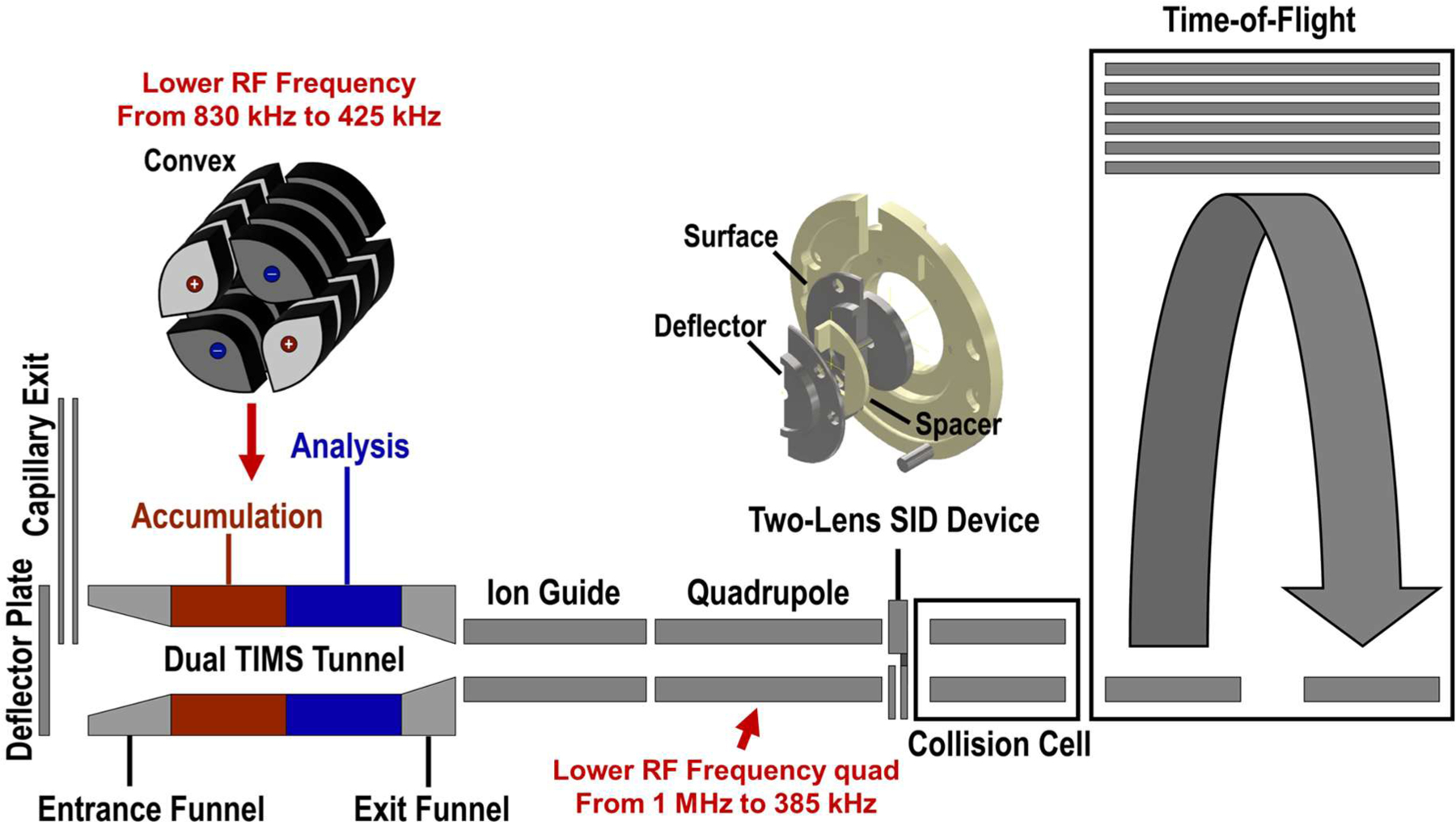
The instrument diagram of the modified Bruker timsTOF Pro. The TIMS cartridge has convex geometry electrodes and is operated at 425 kHz. The quadrupole is operated at 385 kHz to extend the isolation range to 17,000 *m/z*. The 2-lens SID device replaces the entrance lens of the collision cell.

To evaluate the performance of the modified instrument (Figure 1), several model protein complexes, including 53 kDa streptavidin (SA), 64 kDa homotetrameric avidin (AV), 103 kDa concanavalin A (Con A), 147 kDa alcohol dehydrogenase (ADH), 58 kDa cholera toxin B (CTB), 115 kDa C-reactive protein (CRP), 90 kDa his-tagged toyocamycin nitrile hydratase (His-TNH), 801 kDa wild-type GroEL, and 3 MDa T=3 and 4 MDa T=4 HBV capsids, were chosen and are discussed below.^30,35,41,43^ HRas*GTP-SOS-HRas complex was used to evaluate TIMS-Q-SID performance.^44^

### Extending the Mass-to-charge Ratio Range for Protein Complex Ion Mobility Analysis.

Initially, the TIMS-Q-TOF instrument was configured with a concave TIMS cartridge (Figure S4a) operating at 830 kHz RF, which can trap ions up to 6,000 *m/z*.^45^ In the work described here, the TIMS cartridge was changed to a convex electrode geometry (inset of Figure 1 and Figure S4b) coupled with a 425 kHz RF frequency TIMS driver to enhance the trapping efficiency for species up to 38,000 *m/z*. The performance of the TIMS cartridge with convex electrodes was verified with selected model protein complexes, which ranged in mass from 50 – 801 kDa (4,500 – 18,500 *m/z*).

**Figure 2.**
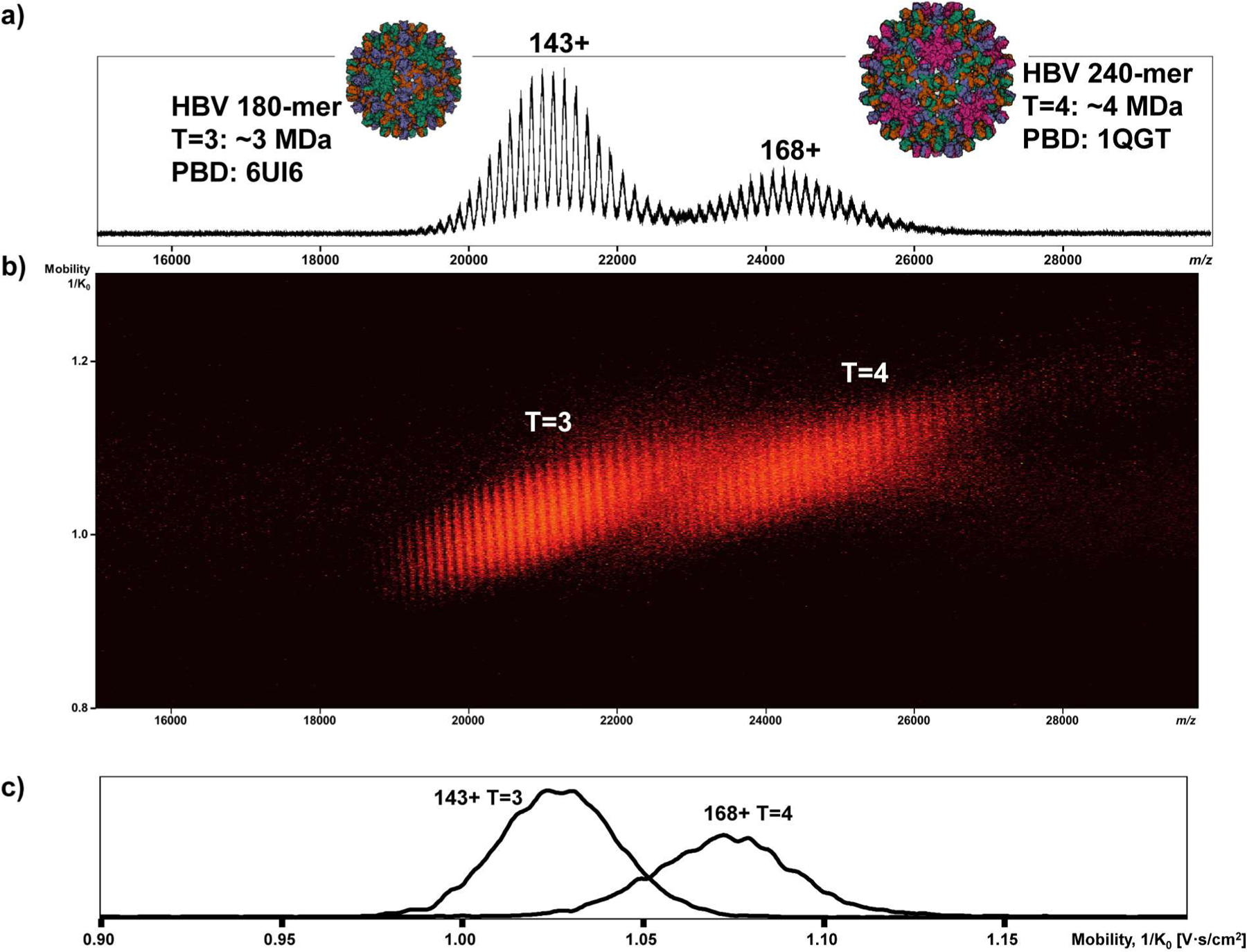
a) The spectrum of ~33 nM 180-mer (T=3, ~3 MDa) and ~25 nM 240-mer (T=4, ~4 MDa) HBV capsids (~ 6 μM per monomer) in 200 mM ammonium acetate. b) The mobiligram of T=3 and T=4 capsids. c) The extracted 1D mobiligram of 143+ T=3 and 168+ T=4. The modified TIMS cartridge trapped both T=3 and T=4 capsids and measured their ion mobilities.

Previously, Fernandez-Lima and co-workers extensively characterized the performance and resolution of the quadrupolar convex TIMS cartridge using proteins and protein complexes and reported their CCSs.^42^ Here, we selected a few model protein complexes to show the reproducibility of this TIMS cartridge and used ESI Tuning Mix as a calibrant for CCS measurements. The different charge states of lower-mass complexes, SA and ADH, were separated with an average mobility peak resolution (^IM^R_p_) of 22 and 29, respectively (Figure S5). The measured CCSs and mobility resolutions of selected protein complexes are shown in Table S4 and S5. To test the effective *m/z* range of this modified TIMS cartridge, lower-mass complexes and 180-mer T=3 and 240-mer T=4 HBV capsids, with masses of ~3 MDa and ~4 MDa, respectively, were investigated.^35,46^ The mobility peaks for different charge states of lower-mass complexes, SA and ADH, were mostly baseline resolved (Figure S5). Figure 2a shows the MS spectrum of T=3 and T=4 HBV. With the modified TIMS cartridge, T=3 and T=4 capsids were trapped successfully during the ion mobility analysis (Figure 2b). The *m/z* peaks of each distribution are partially resolved (T=3, ^*m/z*^R_p_=~172; T=4, ^*m/z*^R_p_=~142), and the charge states of each HBV capsid can be determined. Figure 2c shows the extracted mobiligram of 143+ T=3 (^IM^R_p_= 17) and 168+ T=4 (^IM^R_p_= 15). T=3 and T=4 capsids were also further charge-reduced with TEAA, and the result is shown in Figure S6. We were still able to trap the charge-reduced T=3 and T=4 capsids (28,000 – 39,000 *m/z*), determine their approximate charge state, and separate the overlapped species using this modified TIMS cartridge (Figure S7). The measured CCS values and calculated ^IM^R_p_ of T=3 and T=4 are shown in Table S6.

### Performance of a Two-lens Surface-induced Dissociation Device.

**Figure 3.**
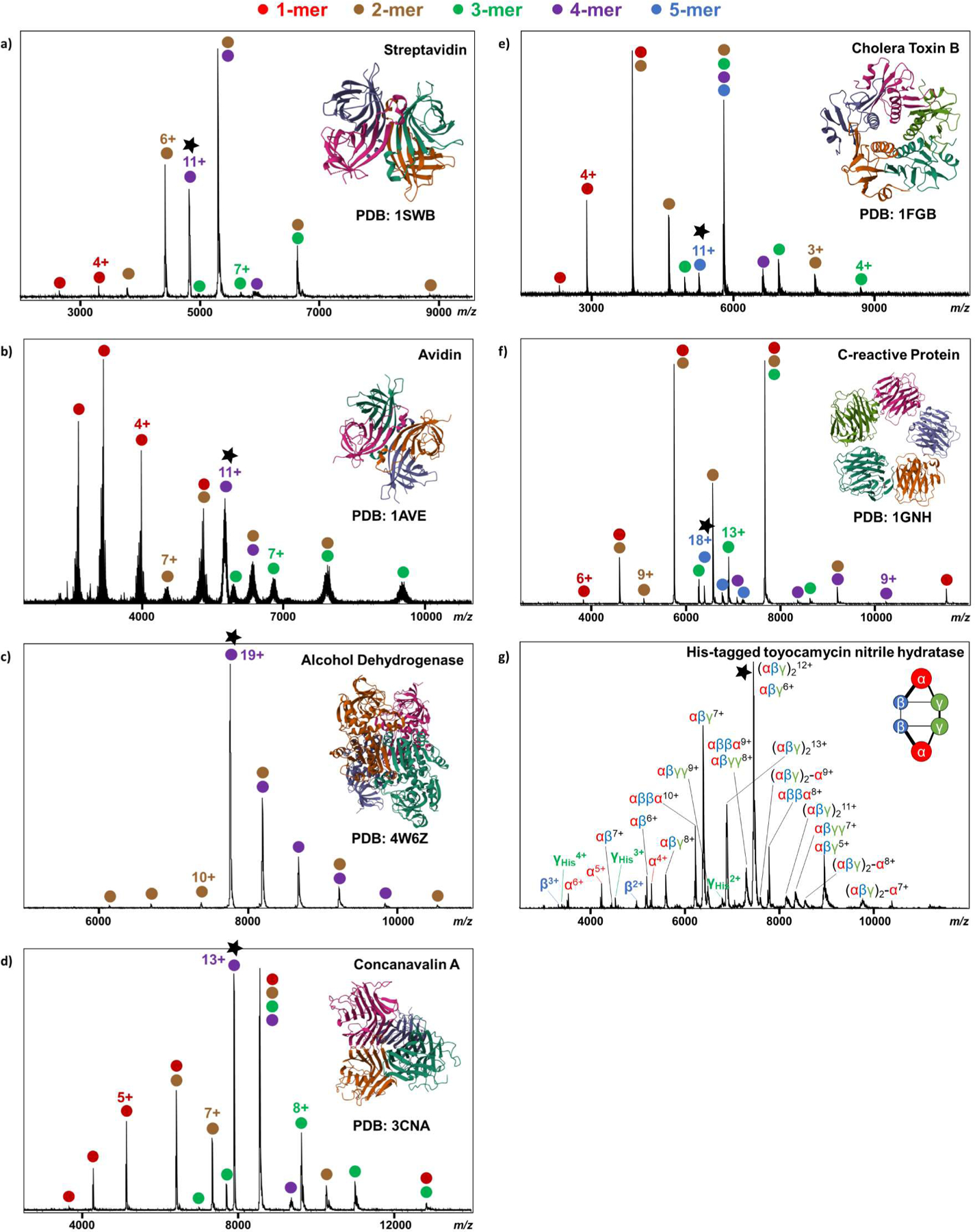
SID spectra of a) 11+ streptavidin at SID 440 eV, b) 11+ avidin at SID 880 eV, c) 19+ alcohol dehydrogenase at SID 3,040 eV, d) 13+ concanavalin A at SID 1,105 eV, e) 11+ cholera toxin B at SID 880 eV, f) 18+ C-reactive protein at SID 1,080 eV, and g) 13+ His-tagged toyocamycin nitrile hydratase at SID 1,170 eV. The results were consistent with previously reported SID data.^30,47,48^ Black stars indicate the isolated precursors. Monomers, dimers, trimers, tetramers, and pentamers are represented by red, brown, green, purple, and blue dots, respectively.

The SID device design on this instrument was inspired by the designs of Snyder et al.^39^ The device is a simplified 2-lens system (inset of Figure 1). The overall width of this device along the ion optical axis is 6 mm, which is the width of the collision cell entrance lens that was removed. The SID surface and extractor were combined into a ring electrode with a 3 mm diameter aperture. The deflector is a half-moon-shaped electrode. Both the surface and deflector were made of 316 stainless steels. An insulated spacer made of polyether ether ketone (PEEK) material was placed between the surface and the deflector to prevent an electrical short circuit. The surface was controlled by an existing spare system voltage on the instrument by entering a command line under service mode, and the deflector was controlled using the voltage typically supplied to the collision cell entrance lens (Focus 2 L3). To verify that the new implementation behaves at least as well as or better than other implementations, using well-known model protein complexes over a wide m/z range is crucial for examining the performance of the installed SID device and results are shown in Figure 3. With the SID device installed, CID can still be performed in the collision cell by setting the two SID lens elements to the same voltage to mimic the standard lens in that position. Charge-reduced SA was used to test the CID performance, and Figure S8 shows a comparison of unaltered and modified TIMS-Q-TOF. The results show that the overall averaged signal-to-noise ratios of products are around 570 before and 465 after the instrument was modified. We did not observe a significant ion transmission loss for CID after the SID installation.

**Figure 4.**
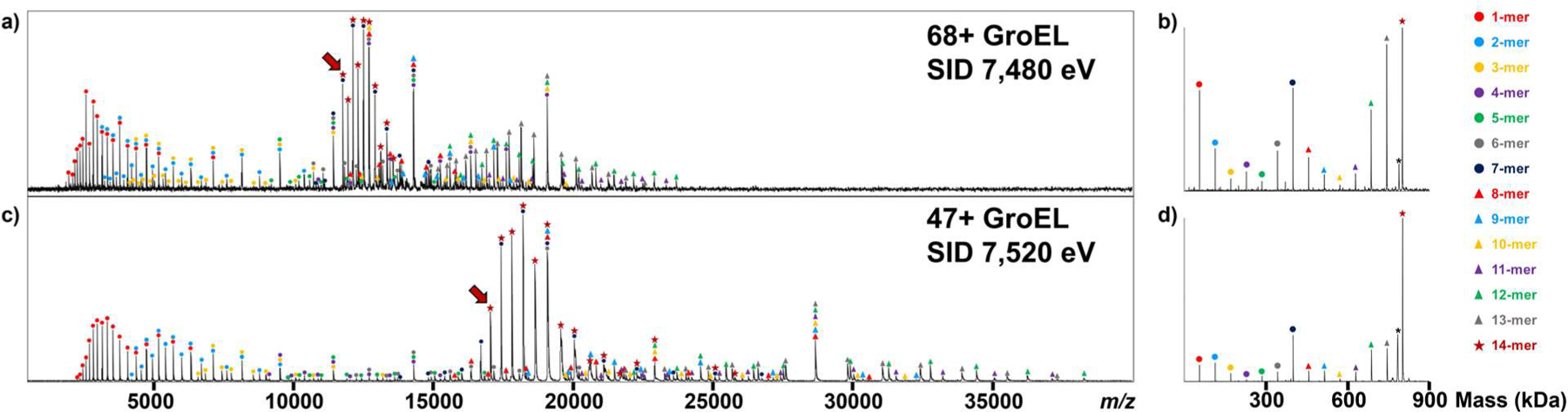
The SID spectra of a) 68+ and c) 47+ GroEL at SID energy 7,480 and 7,520 eV, respectively. The deconvolved mass spectra show the relative abundance of SID products from b) 68+ and d) 47+ GroEL. The red arrows indicate the selected 68+ and 47+ GroEL precursors. The legend shows different subcomplexes of GroEL SID products and their representative symbols. The asterisk represents a deconvolution error. These deconvolution error peaks are assigned from an atypical *m/z* peak distribution (asymmetric peak heights from the centroid peak).

The largest protein complex fragmented here to test the SID performance was 14-mer 801 kDa GroEL, normal charge in 200 mM ammonium acetate and charge-reduced species using TEAA (Figure S9). We isolated 68+ and 47+ GroEL at 11,850 and 16,950 *m/z*, respectively, with an isolation window of 200 *m/z* using quadrupole, and fragmented them using SID. The SID energies were adjusted to 7,520 eV and 7,480 eV, respectively, to compare their results under an approximately equal SID energy level. Figure 4 shows the SID results from both selected ions. The SID products cover the entire *m/z* range selected for analysis. The peaks at high *m/z* are well resolved and less broad than on other platforms we have modified. Fragments corresponding 10- to 13-mers at 30,000 to 40,000 *m/z* were not reported previously for other instrument platforms.^41,49^ After deconvolving the spectrum using UniDec^50^, the results show that, after SID, 47+ GroEL (Figure 4d) has fewer monomers and 13-mers formed, and it is more favorable to produce 7-mers, which reflects a more native-like structure compared to the SID result of 68+ GroEL (Figure 4b).

### Analysis of a complex mixture using the modified TIMS-Q-TOF.

**Figure 5:**
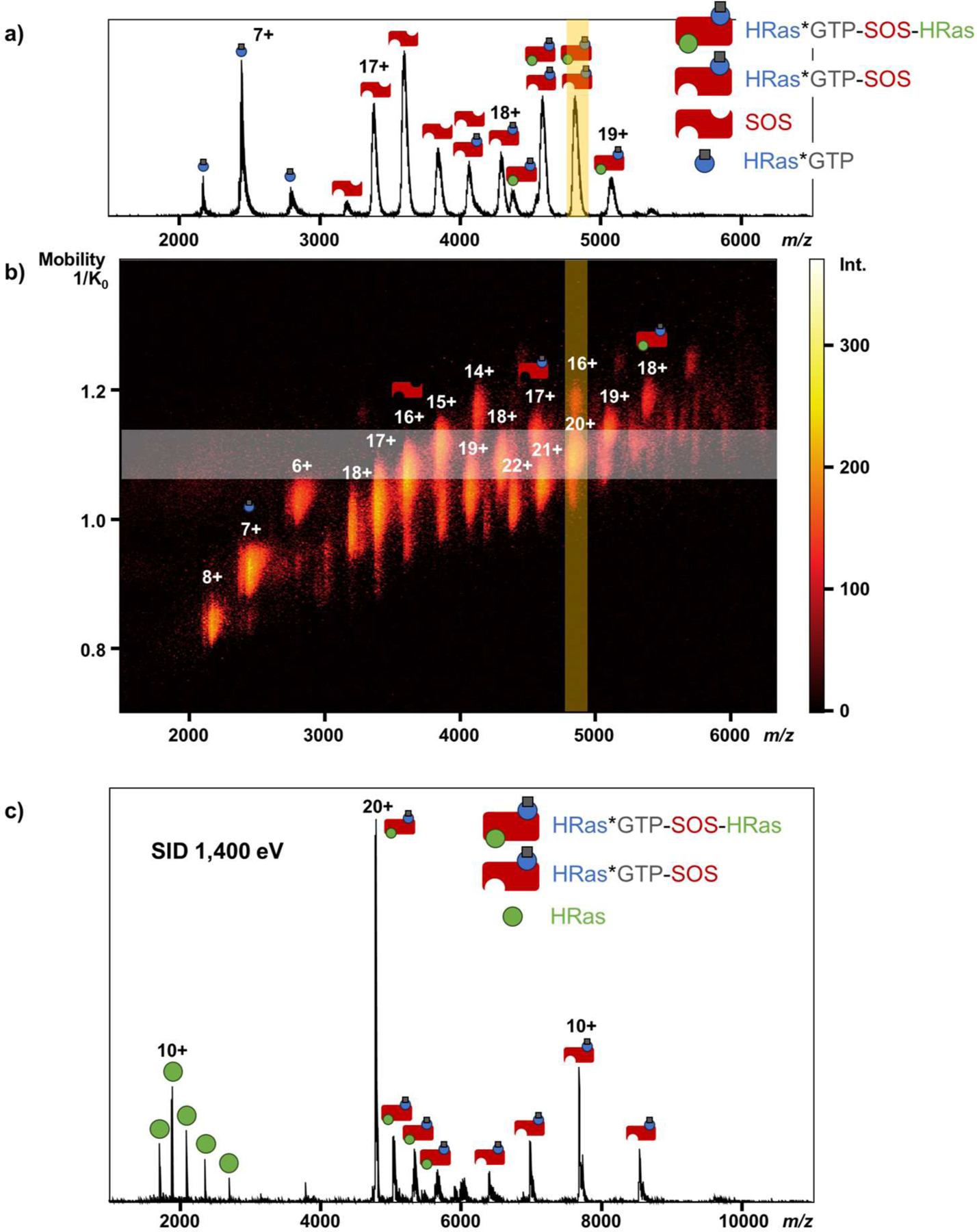
a) The mass spectrum and b) the mobiligram of the HRas-SOS complex. c) The SID result of 20+ HRas*GTP-SOS-HRas at SID 1,400 eV. The shaded yellow area represents the isolation using quadrupoles, while the white area represents the extracting mobility area (1.07 – 1.11 1/K_0_) used to generate the SID spectrum after quadrupole isolating at 4,800 *m/z* with a width of 100 *m/z*.

When the sample is a complex mixture, sometimes the protein complex of interest can overlap with others under the same *m/z*. Using TIMS to separate the mixture by ion mobility (IM) and isolating a narrow *m/z* range of interest before SID makes the identification of SID fragments easier. GTPase, Ras, is a key component of the mitogen-activated protein kinase signaling pathway, cycling between guanosine diphosphate-bound inactive and guanosine triphosphate (GTP)-bound active states.^51^ It forms a complex with a specific guanine nucleotide exchange factor, Son of Sevenless (SOS). Ras*GTP binds at the distal site, which modulates the activity of SOS, while Ras binds at the active site, facilitating nucleotide exchange.^44,52^ To investigate the HRas-SOS complex structure, HRas*GTP and SOS were mixed and ionized and 4 different proteins and protein complexes, HRas*GTP, SOS, HRas*GTP-SOS, and HRas*GTP-SOS-HRas, were observed in the MS1 spectrum (Figure 5a). We were interested in studying the structure of the full HRas*GTP-SOS-HRas complex. However, from the mobiligram shown in Figure 5b, there were always other protein complexes overlapped with HRas*GTP-SOS-HRas in *m/z*. Those overlapped protein complexes, which could not be separated without IM or by narrowing the *m/z* range using quadrupole, were also fragmented when performing MS/MS on HRas*GTP-SOS-HRas, convoluting interpretation of results. Therefore, TIMS was used to separate overlapped species prior to quadrupole isolation and MS/MS to improve the structural analysis of the HRas*GTP-SOS-HRas complex.

To perform SID and characterize the HRas*GTP-SOS-HRas complex, the mixture was first separated using TIMS. We then isolated a narrow *m/z* range of interest, and 16+ HRas*GTP-SOS and 20+ HRas*GTP-SOS-HRas were isolated. These two complexes were then fragmented by SID. After SID, product ions from each precursor were mobility-aligned in the heat map, which is shown in Figure S10. To identify the SID fragments of 20+ HRas*GTP-SOS-HRas, we generated the SID spectrum of 20+ HRas*GTP-SOS-HRas by extracting its mobility peak (Figure 5c). The result shows that HRas*GTP-SOS-HRas dissociates into two species: HRas and HRas*GTP-SOS, and HRas*GTP retains on SOS after SID. This indicates that HRas*GTP binds more strongly with SOS than HRas. It’s also interesting to note that HRas carried about half of the charges from the precursor after being dissociated from the complex, which was not anticipated. Typically, SID fragments carry charges proportional to the mass of the precursor.^27,29,32^ This suggests that HRas undergoes structural rearrangement to depart from the complex, which is similar to results reported for some other complexes.^53^ To understand the effect of GTP on HRas’ structure stability, further study is required. Overall, by combining TIMS separation and quadrupole isolation, we were able to study the structure of HRas*GTP-SOS-HRas using SID without any interference from other complexes in the mixture.

## Conclusions

Here, we demonstrated that with a low RF frequency convex TIMS cartridge, we were able to mobility-separate charge-reduced 3 and 4 MDa T=3 and T=4 HBV capsids. A single charge state of charge-reduced GroEL could be quadrupole-selected at *m/z* 16,200 with a 25 *m/z* isolation window with the low RF frequency quadrupole driver. Also, a 2-lens SID device was able to fragment 801 kDa GroEL and investigate its subunit connectivity. The MS/MS fragments cover a wider *m/z* range under a single set of tuning conditions than previously reported for other instrument platforms. Significantly, the peaks of fragments at high *m/z* are relatively narrow and well resolved, which improves the identification of different subcomplexes. The SID device also replaces the preexisting entrance lens of the collision cell, requiring no hardware modifications and relatively simple installation. SID can also be used as a tool to probe the structural information of ions of interest. The detection limit of this instrument is also outstanding. We were able to perform nMS for HBV capsids in the concentration range of 20 to 50 nM. Moreover, with ion mobility separation prior to the quadrupole isolation and SID fragmentation, when characterizing a specific protein complex from a complex mixture using nMS, we were able to differentiate those SID products from the *m/z* overlapping precursors. The fragments were mobility-aligned with their precursor, which simplifies the interpretation of SID products to study their subcomplex arrangement and connectivity.

## Supplementary Material

SI

Additional materials, instrument parameters, SID operation, collision cross-sections of selected protein complexes, mobility peak resolution of selected protein complexes, supporting figures of the mass spectra of isolated 49+ GroEL, diagram of the TIMS cartridge, comparison of different average times of charge-reduced cholera toxin B SID spectra, electrode geometry of concave and convex TIMS cartridges, mass spectra of streptavidin and alcohol dehydrogenase and their extracted mobility peaks, charge-reduced T3 and T4 HBV mass spectrum, heat map and mobiligrams, MS spectra of normal charge and charge-reduced GroEL, CID spectra of charge-reduced streptavidin on unaltered and modified timsTOF Pro, and the SID heat map of 16+ HRas*GTP-SOS and 20+ HRas*GTP-SOS-HRas.
