# Supplementary material for: Adapting a trapped ion mobility spectrometry-Q-TOF for high m/z native mass spectrometry and surface-induced dissociation
Source: Anal Chem. Author manuscript; Available in PMC 2026 Apr 7. (PMC13054468; doi:10.1021/acs.analchem.4c03557)
Supplement: SI [file NIHMS2158746-supplement-SI.pdf]

Supporting Information

Adapting a trapped ion mobility spectrometry-Q-TOF for high *m/z* native mass spectrometry and surface-induced dissociation

Yu-Fu Lin,[1,2] Benjamin J. Jones,[3] Mark E. Ridgeway,[3] Erin M. Panczyk,[3] Arpad Somogyi,[1,2] Desmond A. Kaplan,[4] Ila Marathe, [1,2] Sangho Yun,[6] Karen A. Kirby,[5] Stefan G. Sarafianos,[5] Arthur D. Laganowsky,[6] Melvin A. Park,*[3] Vicki H. Wysocki*[1,2]

1. Department of Chemistry and Biochemistry, The Ohio State University, Columbus, OH 43210, United States

2. Native MS Guided Structural Biology Center, The Ohio State University, Columbus, OH 43210, United States

3. Bruker Daltonics Inc., Billerica, MA 01821, United States

4. KapScience LLC, Tewksbury, MA 01876, United States

5. Center for ViroScience and Cure, Laboratory of Biochemical Pharmacology, Department of Pediatrics, Emory University School of Medicine and Children's Healthcare of Atlanta, Atlanta, GA 30307 United States

6. Department of Chemistry, Texas A&M University, College Station, TX 77840, United States

*Corresponding authors: wysocki.11@osu.edu and Mel.Park@bruker.com

Table of Contents

A. Materials 2

B. SID operation 2

C. Additional tables 3

D. Additional Figures 8

E. Reference 14

A. Materials

Low concentration Tuning Mix (G24221A) was purchased from Agilent Technologies (Santa Clara, CA) as a standard for mass and mobility calibration. Ammonium acetate, triethylammonium acetate (TEAA), streptavidin (Thermo Pierce 21125), cholera toxin B (Sigma C9903), avidin (Sigma A9275), concanavalin A (Sigma C2010), and alcohol dehydrogenase (Sigma A7011) were purchased from Sigma-Aldrich (St. Louis, MO). C-reactive protein (EMD Millipore 236608) was obtained from Lee Biosolutions (Maryland Heights, MO). The Bandarian lab at the University of Utah expressed and purified his-tagged toyocamycin nitrile hydratase, which has been reported previously.[1] Wild-type GroEL was expressed and purified by the Hayes Rye Group at Texas A&M University (College Station, TX).[2] T=3 and T=4 hepatitis B virus (HBV) capsids were purified and assembled as previously described and obtained from Karen Kirby and Stefan Sarafianos at Emory University (Atlanta, GA).[3] The HRaswt-GTP and Son of Sevenless (SOS) protein were expressed and purified as published previously and obtained from the Laganowsky lab at Texas A&M University-College Station.[4]

B. SID operation

When performing SID experiments, the surface was set to 35.5 V and controlled using an instrument command line. The “Collision Cell bias” was 25.5 V, 10 V lower than the surface, to improve ion extraction after surface collisions. The deflector electrode voltage was more positive when deflecting ions into the surface. After testing the SID device with the model protein complex, 53 kDa streptavidin, and adjusting parameters, Equation 1 summarizes the calculation of deflector voltage to perform SID 10 V on TIMS-Q-TOF.

Equation 1

$$V_{D-SID10} = 1.5 \times V_{SID} + V_{L3} + V_{SID} + 5$$

Where $V_{D\text{-}SID10}$ is the deflector voltage of SID 10 V, V_{SID} is the target SID potential, and V_{L3} is the default “Focus 2 L3” value (26.2 V). After the deflector voltage of SID 10 V was defined, the deflector voltages for other SID energy potentials were adjusted relative to $V_{D\text{-}SID10}$. In general, the deflector voltage increased by 2 V for every 5 V of SID potential starting from SID 10 V. Parameters for each SID potential are listed in Table S2. The deflector voltages were also adjusted based on the “Deflector voltage (Focus 2 L3)” listed in Table S2 depending on the protein complexes’ size. Higher *m/z* protein complex ions require higher deflector voltage to get better SID efficiency. Using GroEL as an example, we added 30 volts higher than the deflector voltage listed in the table to produce more SID products and minimize the contamination of products produced by CID. The “Collision Energy”, which is the CID potential, was 10.0 V higher than the desired SID voltage to accelerate ions prior to deflecting to the surface. With this design, only three parameters were required to control SID, and one of them was a fixed value (surface voltage), which simplifies the SID operation. When SID was performed, all the electrode voltages before the surface electrode were elevated by “Collision Energy” relative to the first electrode of the collision cell. The “Quadrupole Bias Exit” is the last electrode of the quadrupole before the SID surface electrode, which is also elevated by “Collision Energy”. The SID potential is defined by the difference between “Quadrupole Bias Exit” voltage and surface voltage (Table S3).

C. Additional tables

Table S1. Default instrument parameters and general instrument parameters for native mass spectrometry of proteins or complexes of a given protein mass. The default setting is used for optimization, calibration, and maintenance using ESI Tuning Mix. [a]Transfer Time and Collision Gas Flow Rate were tuned based on protein mass. [b]The Collision Gas Flow Rate was set to 35.0 % for ESI Tuning Mix. [c]Because this is a prototype TIMS cartridge, the default TIMS IN Pressure is to be determined.

Instrument parameter	System default setting	Protein mass (kDa)		
		120 kDa or below	120 to 350 kDa	350 kDa or above
Capillary (V)	800	800	800	1,200
Dry Gas (L/min)	3.0	4.0	4.0	4.0
Dry Temp (°C)	150	90	90	90
$1/K_0$ Start (Vs/cm^2)	0.78	0.60	0.60	0.60
$1/K_0$ End (Vs/cm^2)	1.60	1.50	1.50	1.50
Accumulation Time (ms)	10.0	100.0	100.0	100.0
Ramp Tims (ms)	93.1	1,000.0	1,000.0	1,000.0
Duty Cycle (%)	10.74	10.00	10.00	10.00
isCID Energy (eV)	10.0	10.0	10.0	10.0
Funnel 1 RF (V_{pp})	350.0	300.0	300.0	300.0
Funnel 2 RF (V_{pp})	400.0	600.0	600.0	600.0
Multipole RF (V_{pp})	500.0	800.0	800.0	800.0
Ion Energy (V)	5.0	5.0	5.0	5.0
Collision Cell In (V)	300.0	150.0	150.0	150.0
Collision RF (V_{pp})	2000.0	2,800.0	2,800.0	2,800.0
Collision Energy (V)	10.0	10.0	10.0	10.0
Pre Pulse Storage (µs)	10.0	35.0	35.0	35.0
Transfer Time (µs)[a]	110.0	140.0	320.0	520.0
Collision Gas Flow Rate (%)[a]	35.0	35.0[b]/88.0	88.0	88.0
$\Delta t1$ (V)	-20.0	-20.0	-20.0	-20.0
$\Delta t2$ (V)	-120.0	-90.0	-90.0	-90.0
$\Delta t3$ (V)	70.0	70.0	70.0	70.0
$\Delta t4$ (V)	100.0	70.0	70.0	70.0
$\Delta t5$ (V)	0.0	0.0	0.0	0.0
$\Delta t6$ (V)	100.0	10.0	10.0	10.0
TIMS IN Pressure (mbar)	N/A[c]	2.2	2.2	2.2

Table S2. SID parameters for SID 10 to 200 V. [a]Collision Energy is the setting in otof control for CID potential.

SID (V)	Collision Energy (eV) [a]	Deflector voltage (Focus 2 L3)
10	20	56.2
20	30	60.2
30	40	64.2
40	50	68.2
50	60	72.2
60	70	76.2
70	80	80.2
80	90	84.2
90	100	88.2
100	110	92.2
110	120	96.2
120	130	100.2
130	140	104.2
140	150	108.2
150	160	112.2
160	170	116.2
170	180	120.2
180	190	124.2
190	200	128.2
200	210	132.2

Table S3. SID device parameters for flythrough and SID 50V. [a]Collision Energy is the setting in otof control for CID potential, [b]Quadrupole Bias Exit changes relatively to Collision Energy.

SID parameter	Flythrough	SID 50 V
Collision Energy (eV)[a]	10.0	60.0
Quadrupole Bias Exit (V)[b]	35.5	85.5
Deflector Voltage (V)	26.2	68.2
Surface Voltage (V)	26.2	35.5

Table S4. Collision cross-section of selected protein complexes. Abbreviation used: ^{tTP}CCS = Averaged collision cross-sections (CCSs) from three replicates measured by the modified instrument using nitrogen gas, % STDV = Standard deviation percentage, and ^{Lit}CCS = Literature CCSs measured using linear ion mobility cell with nitrogen gas.

Protein complex	Charge State	^{tTP}CCS (Å^2)	% STDV	^{Lit}CCS (Å^2)
Avidin	10	3834	0.69	–
	11	3865	0.60	3960
	12	3886	0.53	3960
Concanavalin A	13	5502	1.24	–
	14	5563	1.03	5840
	15	5607	1.73	5850
C-reactive Protein	16	6669	0.51	6880
	17	6716	0.41	6900
	18	6760	0.37	6920
Glutamate dehydrogenase	25	12036	0.42	12450
	26	12200	0.83	12440
	27	12157	0.32	12430
	28	12207	0.47	12430

Table S5. Mobility peak resolution of selected protein complexes. The TIMS measures the analyte's mobility and is reported as the invert of K_0 ($1/K_0$). The ion mobility single peak resolution is defined as $(1/K_0)/\Delta(1/K_0)$. The ion mobility single peak resolutions vary from each charge state due to a distribution of unresolved conformations.[5] Abbreviation used: ${}^{IM}R_p$ = Ion mobility single peak resolution.

Protein complex	Charge State	${}^{IM}R_p$
Streptavidin	9	27
	10	18
	11	22
Cholera toxin B	9	41
	10	27
	11	26
	12	38
Avidin	10	35
	11	25
	12	23
	13	33
Concanavalin A	12	43
	13	37
	14	26
	15	45
Alcohol dehydrogenase	16	37
	17	28
	18	24
	19	27
GroEL	46	44
	47	40
	48	32
	49	36

Table S6. Collision cross-section of hepatitis B virus T=3 and T=4 capsids and the mobility peak resolution from selected charge states. Collision cross-sections were measured using nitrogen gas. The TIMS measures the analyte's mobility and is reported as the invert of K_0 ($1/K_0$). The ion mobility single peak resolution is defined as $(1/K_0)/\Delta(1/K_0)$. Abbreviation used: ^{tTP}CCS = Averaged collision cross-sections (CCSs) by the modified instrument using nitrogen gas and $^{IM}R_p$ = Ion mobility single peak resolution.

HBV capsids	Charge State	^{tTP}CCS (Å^2)	$^{IM}R_p$
Normal-charged T=3	94	50592	18
	95	50729	18
	96	50816	18
	97	50857	18
Charge-reduced T=3	142	55566	16
	143	55699	17
	144	55701	17
	145	55876	17
Normal-charged T=4	109	61289	13
	110	61479	14
	111	61406	14
	112	61697	14
Charge-reduced T=4	166	67810	16
	167	68007	15
	168	68175	16
	169	68158	14

D. Additional Figures

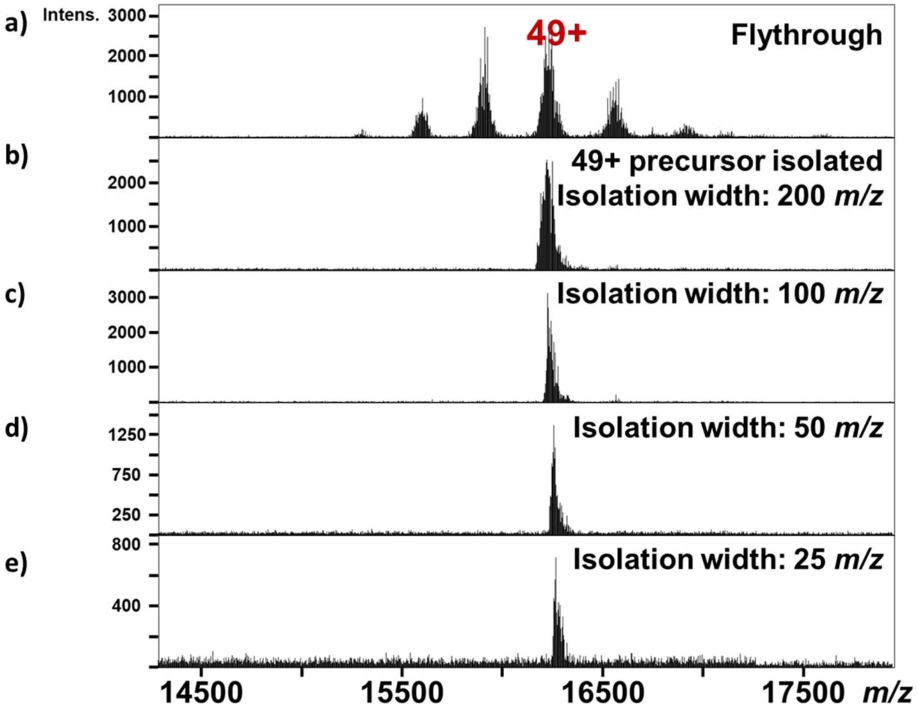

Figure S1. a) The mass spectrum of charge reduced GroEL. The 49+ charge state was isolated using a low RF frequency quadrupole driver with an isolation width of b) 200, c) 100, d) 50, and e) 25 *m/z*.

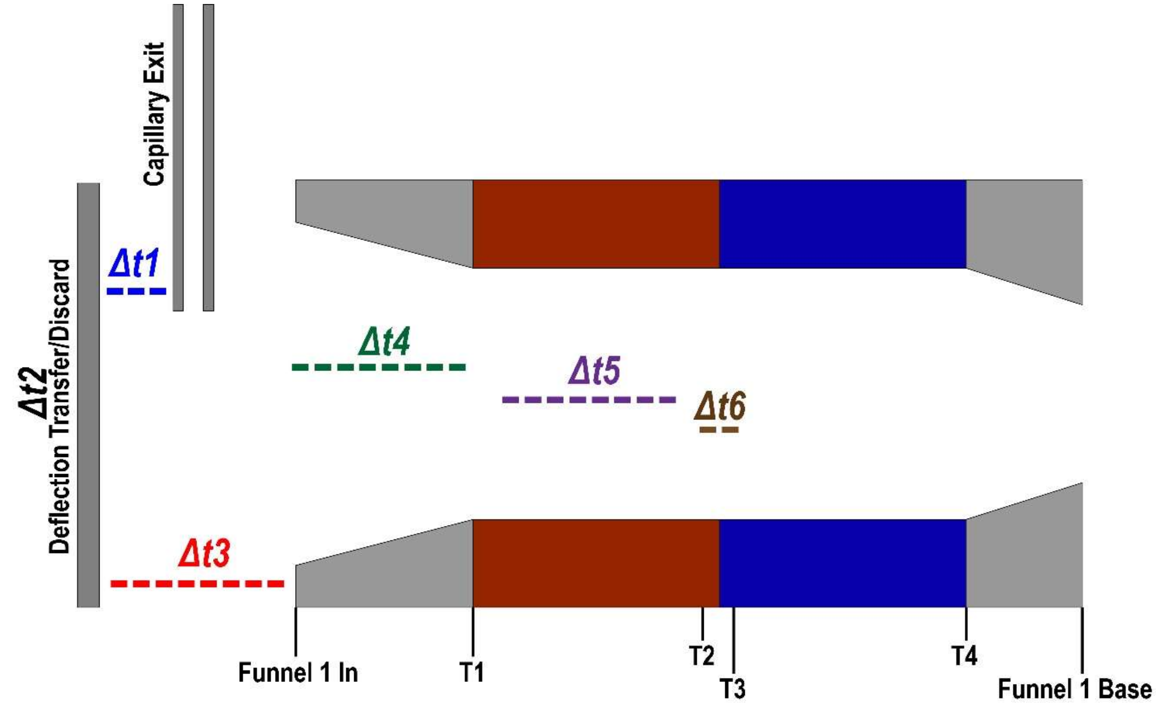

Figure S2. The diagram of the TIMS device with "Delta Values" labeled on otofControl 6.2 and their corresponding electrodes. *Δ*t1 (blue dash line): "Deflection Transfer" to "Capillary Exit", *Δ*t2 (black): "Deflection Transfer" to "Deflection Discard", *Δ*t3 (red dash line): "Funnel 1 In" to "Deflection Transfer", *Δ*t4 (green dash line): "Accumulation Trap (T1)" to "Funnel 1 in", *Δ*t5 (purple dash line): "Accumulation Exit (T2)" to "Accumulation Transfer (T1)", and *Δ*t6 (brown dash line): "Ramp Start (T3)" to "Accumulation Exit (T2)".

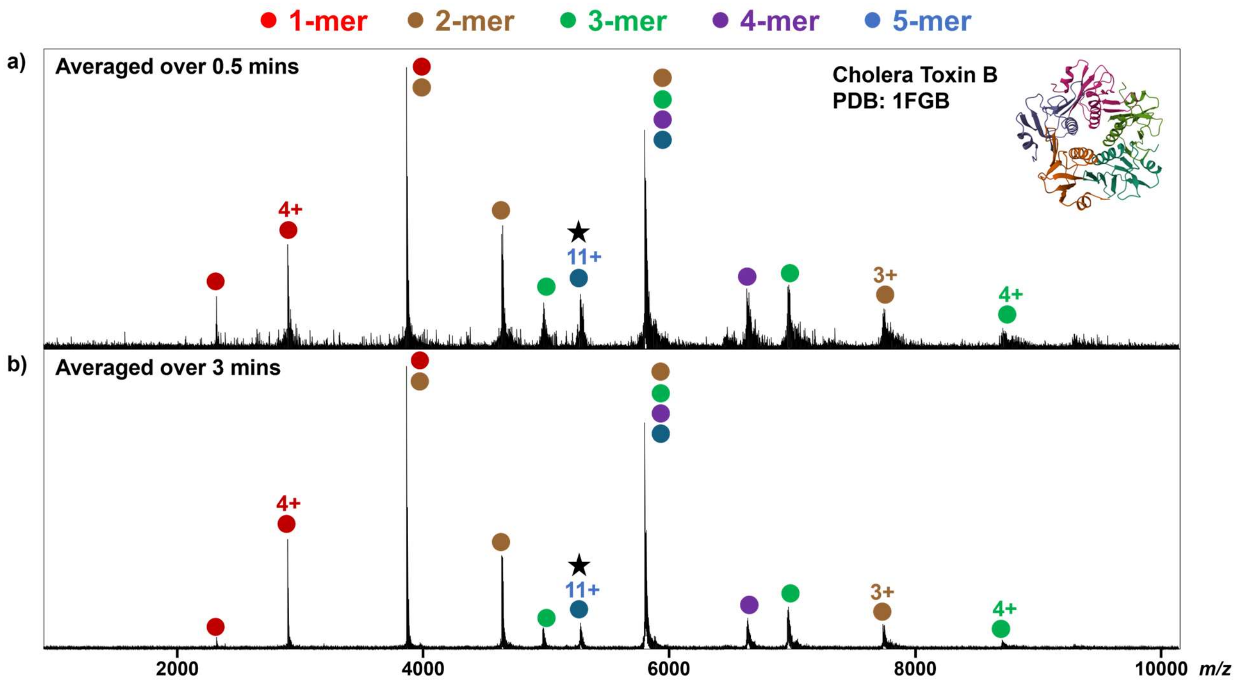

Figure S3. SID spectra of charge-reduced cholera toxin B averaged over a) 0.5 and b) 3 minutes at SID 880 eV. Where averaging the data with longer time, the spectrum has a higher signal-to-noise ratio. Therefore, a longer requisition time provides better quality of the SID spectrum. Black stars indicate the isolated precursors. Monomers, dimers, trimers, tetramers, and pentamers are represented by red, brown, green, purple, and blue dots, respectively.

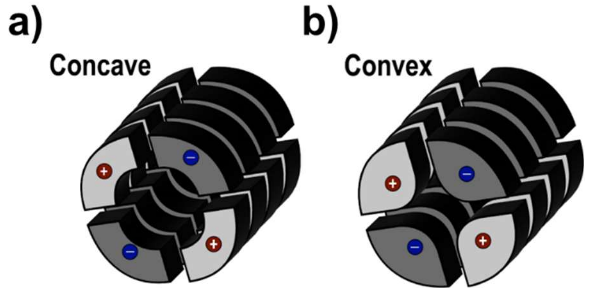

Figure S4. The electrode geometry of a) concave and b) convex TIMS cartridges. The convex geometry provides a stronger pseudopotential electric field, improving the trapping efficiency of high *m/z* ions.[6]

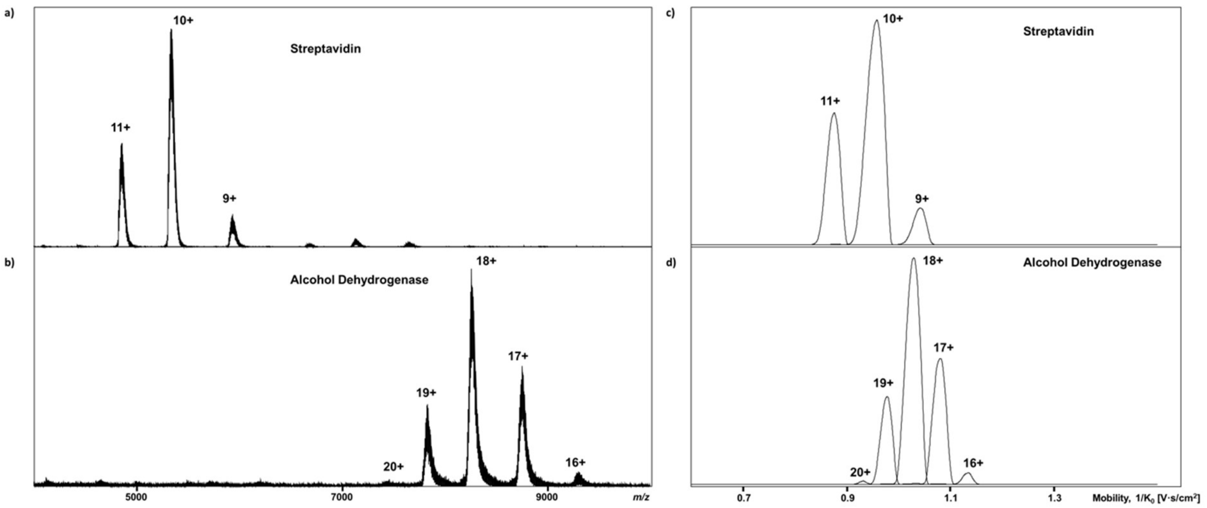

Figure S5. Mass Spectra for charge reduced a) streptavidin and b) alcohol dehydrogenase and extracted mobility peaks of c) 9+ – 11+ streptavidin and d) 16+ – 20+ alcohol dehydrogenase.

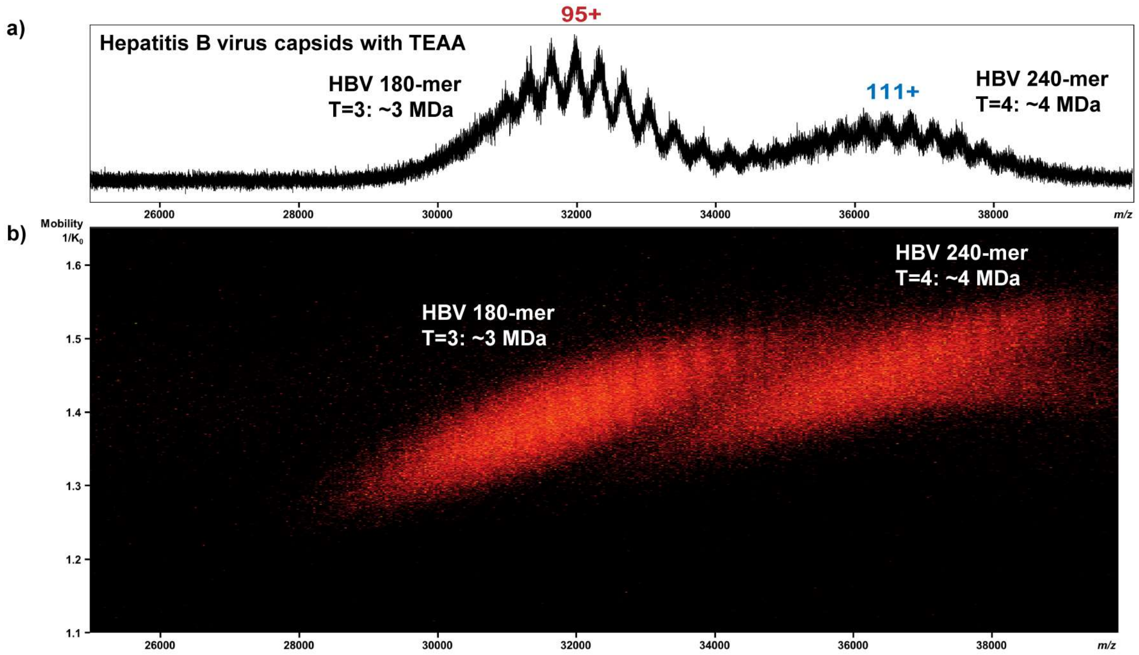

Figure S6. a) The spectrum of ~27 nM 180-mer (T=3, ~3 MDa) and ~20 nM 240-mer (T=4, ~4 MDa) HBV capsids (~ 6 µM per monomer) in 160 mM ammonium acetate, 40 mM triethylammonium acetate (TEAA). b) The mobiligram of T=3 and T=4 capsids. Both T=3 and T=4 capsids were trapped and further separated after charge reduction using TEAA.

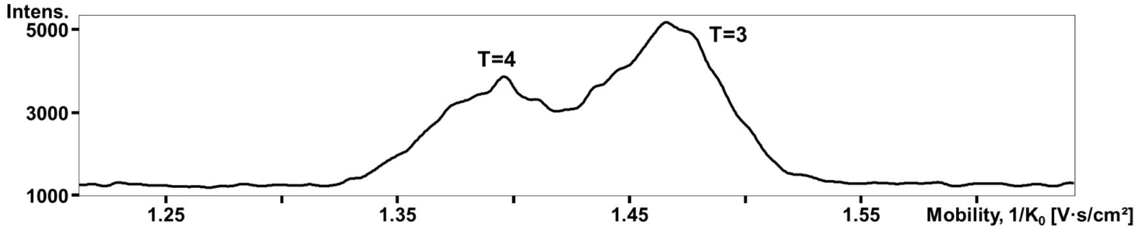

Figure S7. The extracted mobilities of T=3 and T=4 capsids from the overlapped *m/z* region (34,000 – 34,300 *m/z*).

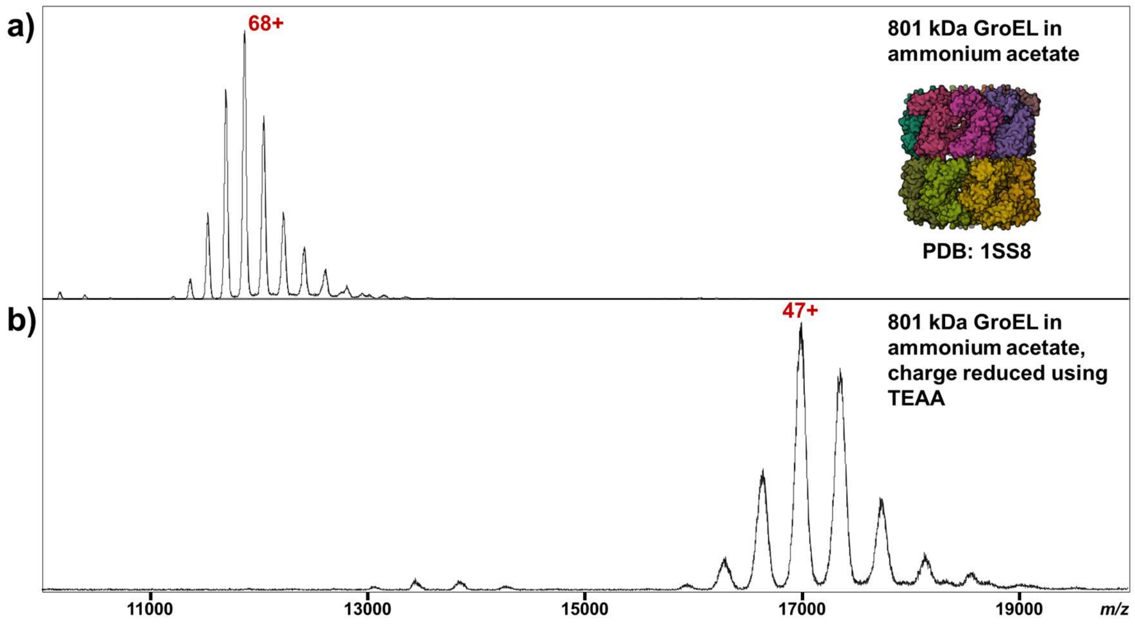

Figure S8. GroEL mass spectra in a) 200 mM ammonium acetate and b) charge reduced with triethylammonium acetate.

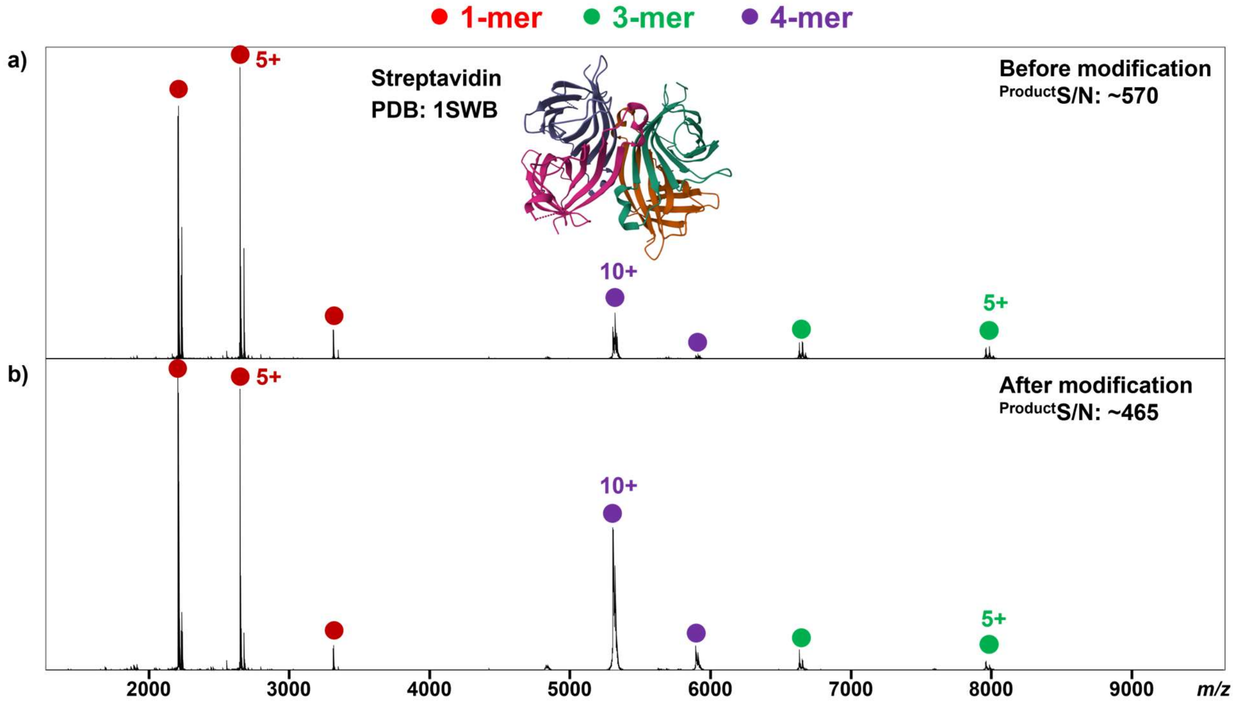

Figure S9. CID spectra of charge-reduced streptavidin a) on an unaltered timsTOF Pro in the OSU Campus Chemical Instrument Center and b) CID on the instrument described in this paper after the addition of SID. To compare the CID performance and post-CID ion transmission, the entire charge state distribution of streptavidin was dissociated using CID before and after the instrument was modified because the

charge-reduced streptavidin *m/z* exceeded the isolation range of the commercial instrument configuration (experiment performed before the quadrupole RF generator modification). ^{Product}S/N indicates the overall average S/N of products. Monomers, trimers, and tetramers are represented by red, green, and purple dots, respectively.

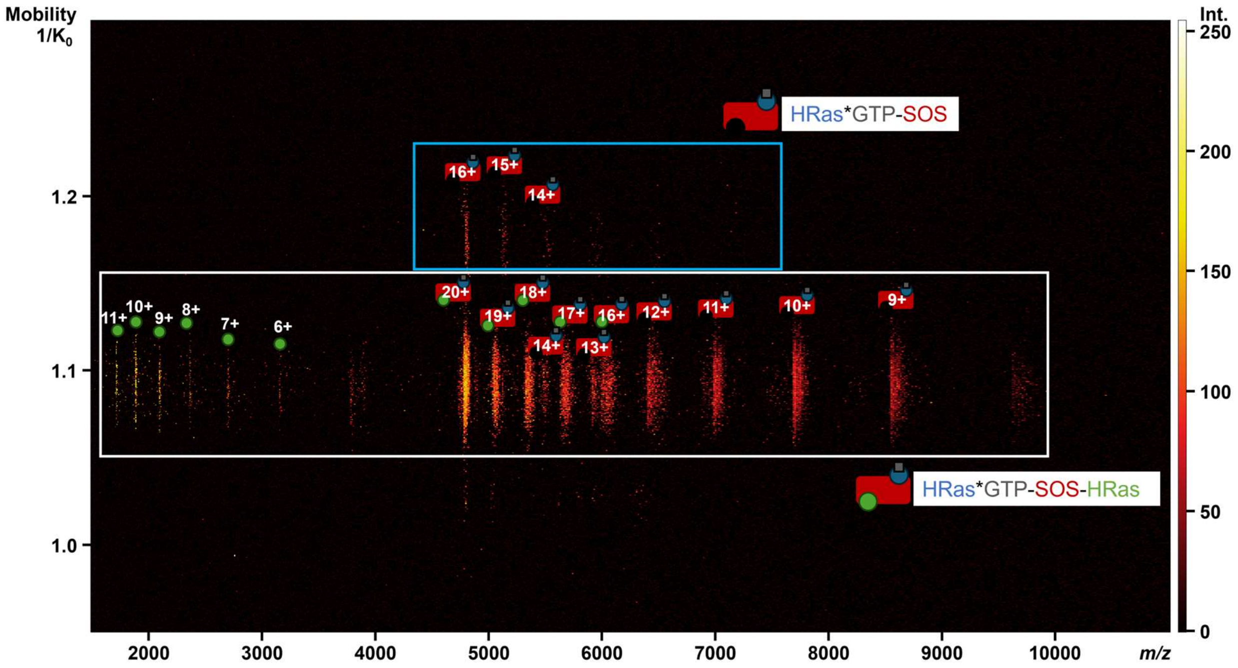

Figure S10. The SID heat map of 16+ HRas*GTP-SOS and 20+ HRas*GTP-SOS-HRas. Product ions were mobility-aligned from their precursor. 20+ HRas*GTP-SOS-HRas (white box) was fragmented into subcomplexes via SID, but 16+ HRas*GTP-SOS (blue box) only reduced charge after SID.

E. Reference

(1) Song, Y.; Nelp, M. T.; Bandarian, V.; Wysocki, V. H. Refining the Structural Model of a Heterohexameric Protein Complex: Surface Induced Dissociation and Ion Mobility Provide Key Connectivity and Topology Information. *ACS Cent. Sci.* **2015**, *1* (9), 477–487. https://doi.org/10.1021/acscentsci.5b00251.

(2) Weaver, J.; Jiang, M.; Roth, A.; Puchalla, J.; Zhang, J.; Rye, H. S. GroEL Actively Stimulates Folding of the Endogenous Substrate Protein PepQ. *Nat Commun* **2017**, *8*, 15934. https://doi.org/10.1038/ncomms15934.

(3) Huber, A. D.; Pineda, D. L.; Liu, D.; Boschert, K. N.; Gres, A. T.; Wolf, J. J.; Coonrod, E. M.; Tang, J.; Laughlin, T. G.; Yang, Q.; Puray-Chavez, M. N.; Ji, J.; Singh, K.; Kirby, K. A.; Wang, Z.; Sarafianos, S. G. Novel Hepatitis B Virus Capsid-Targeting Antiviral That Aggregates Core Particles and Inhibits Nuclear Entry of Viral Cores. *ACS Infect. Dis.* **2019**, *5* (5), 750–758. https://doi.org/10.1021/acsinfecdis.8b00235.

(4) Yun, S. D.; Scott, E.; Moghadamchargari, Z.; Laganowsky, A. 2′-Deoxy Guanosine Nucleotides Alter the Biochemical Properties of Ras. *Biochemistry* **2023**, *62* (16), 2450–2460. https://doi.org/10.1021/acs.biochem.3c00258.

(5) Liu, F. C.; Cropley, T. C.; Ridgeway, M. E.; Park, M. A.; Bleiholder, C. Structural Analysis of the Glycoprotein Complex Avidin by Tandem-Trapped Ion Mobility Spectrometry–Mass Spectrometry (Tandem-TIMS/MS). *Anal. Chem.* **2020**, *92* (6), 4459–4467. https://doi.org/10.1021/acs.analchem.9b05481.
(6) Jeanne Dit Fouque, K.; Garabedian, A.; Leng, F.; Tse-Dinh, Y.-C.; Ridgeway, M. E.; Park, M. A.; Fernandez-Lima, F. Trapped Ion Mobility Spectrometry of Native Macromolecular Assemblies. *Anal. Chem.* **2021**, *93* (5), 2933–2941. https://doi.org/10.1021/acs.analchem.0c04556.
